# Chronic heat stress regulates the relation between heat shock protein and immunity in broiler small intestine

**DOI:** 10.1038/s41598-020-75885-x

**Published:** 2020-11-02

**Authors:** Sharif Hasan Siddiqui, Darae Kang, Jinryong Park, Mousumee Khan, Kwanseob Shim

**Affiliations:** 1 Department of Animal Biotechnology, College of Agriculture and Life Sciences, Jeonbuk National University, Jeonju, 54896 Republic of Korea; 2 Department of Biomedical Sciences and Institute for Medical Science, Jeonbuk National University Medical School, Jeonju, 54907 Republic of Korea; 3 Department of Agricultural Convergence Technology, Jeonbuk National University, Jeonju, 54896 Republic of Korea

**Keywords:** Biotechnology, Animal biotechnology

## Abstract

Chronic heat stress is considered to decrease the immune functions which makes negative effect on broiler growth performance. Here, we investigated the relationship between chronic heat stress, growth performance, and immunity in the small intestine of broilers. The study included two groups (control and heat stressed group) with eight replications per group. Ten broilers of 20-day aged were allocated in each replication. On day 35, the treatment group was subdivided into two groups based on their body weights (heavy and low body weight). Although, there was only the control and treatment group on day 28. The growth performance decreased and expression of heat shock protein 70 (HSP70), HSP60, and HSP47 increased on days 28 and 35 in the chronic heat stress group as compared with those in the control group. The expression levels of HSPs were significantly higher in the low body weight group than in the control group. The genes HSP70 and HSP60 were significantly associated with pro- and anti-inflammatory cytokines in the small intestine of the broilers of the treatment group. Thus, HSP70 and HSP60 activated the adaptive immunity in the small intestines of the broilers from the treatment group to allow adaptation to chronic heat stress environment.

## Introduction

High environmental temperature generates heat waves, which ultimately induce heat stress^1^. Previous study defines, heat stress is a condition, while an animal cannot dissipate their body temperature in the surrounding due to comparative higher temperature in the environment^2^. In addition, the heat stress is classified into acute and chronic stress based on heat exposure duration, whereas acute heat stress refers to short period heat exposure and chronic heat stress refer to extended periods of heat exposure^3^. Furthermore, the summer season is extending due to climate changes. Therefore, heat stress has raised global concerns for the poultry industry, given its adverse effects on the functions of different organs as well as on the growth performance of broilers^4–6^. In response to heat stress, loses intestinal integrity, as well as decrease nutrients digestion and absorption, and the ultimate result is body weight reduced^7^. Although, the Ross broiler contained comparatively longer small intestine^8^. Heat stress even alters the appearance of the small intestine^9^ as well as negatively impacts on the immune system^10^ by triggering the expression of heat shock proteins (HSPs), and, thereby leading to reduce energy metabolism^11^ and suppress broiler growth performance.

HSPs are well known for its relation in response to heat stress in all organisms. Heat stress induces the mRNA as well as protein expression of heat-inducible HSPs. These HSPs identify unfolded or misfolded proteins and correct their protein structure or prevent improper protein folding^12^. Researchers classified HSPs based on their molecular weights into HSP100, HSP90, HSP70, HSP60, HSP40, and the small HSP families^13,14^. A recent study demonstrated the significant cytoprotective role of HSP70 in the intestinal epithelium as well as its function in the strengthening of the intestinal barrier^15^. Furthermore, HSP70 is also able to maintain and stabilise the intestinal tight junction^16^ and generate a strong intestinal barrier in the ileum of stressed animals^15^. Furthermore, HSP60 is expressed in the cytoplasm of the villus and crypt epithelial cells of the small intestine^17^, which is to protect the cells from different stress by regulating mitochondrial protein folding process^18^. Overexpression of HSP60 has been associated with the inflammation of intestinal epithelial cells^19^ and has been reported to play protective roles in the intestinal epithelial recovery^14^. On the other hand, HSP47 found in the endoplasmic reticulum is involved in cellular procollagen folding^20^. An inflamed intestine is characterised by intestinal fibrosis^21^, which is regulated through the immediate induction of HSP47 expression^22,23^.

Chronic heat stress responsible for poor growth rate as well as a weak immune system of the animal's body^24,25^. However, in different stress conditions, Pro- and anti-inflammatory cytokines secrete from different immune cells and play a pivotal role to determine the immune status of an organism^26^. Generally, Pro-inflammatory cytokines mediate inflammatory damage, whereas anti-inflammatory cytokines ameliorate inflammation and stimulate the healing process^27,28^. Interleukin (IL)-10 is a crucial anti-inflammatory cytokine involved in the inflammatory response. Previous studies have shown that IL-10 is one of the most important cytokines associated with several pathophysiological conditions^29^, wherein it inhibits the production of pro-inflammatory cytokines^30^. On the contrary, tumor necrosis factor (TNF)-α is a pro-inflammatory cytokine, which widely studied animal models of sepsis^31^. Interestingly, IL-6 possesses both pro- and anti-inflammatory properties, therefore, this interleukin participates in both inflammation and metabolic processes^32^. Moreover, IL-6 involves the modification of the tight junctions of the intestine^33^, whereas TNF-α increases the intestinal permeability^34^. Although, IL-10 inhibits TNF-α expression in inflamed organs to facilitate the development of adaptive immunity in humans^35,36^.

Considering this mentioned circumstances, we hypothesised that chronic heat stress declined the immune function and contributed a negative effect on the growth performance of broilers. Till now, no previous study has evaluated the relationship between chronic heat stress and adaptive immunity in the small intestine of broiler. Therefore, here we investigated the effects of chronic heat stress on the adaptive immunity of different parts of broiler small intestine. We found that broilers activated the adaptive immunity in response to chronic heat stress. We observed that heavy-body-weight broilers exhibited better heat tolerance capacity than low-body-weight broilers owing to their strong immunity. We even proposed a scheme to highlight the relationship between HSPs and pro- and anti-inflammatory cytokines.

## Results

### Effects of chronic heat stress on growth performance

**Figure 1:**
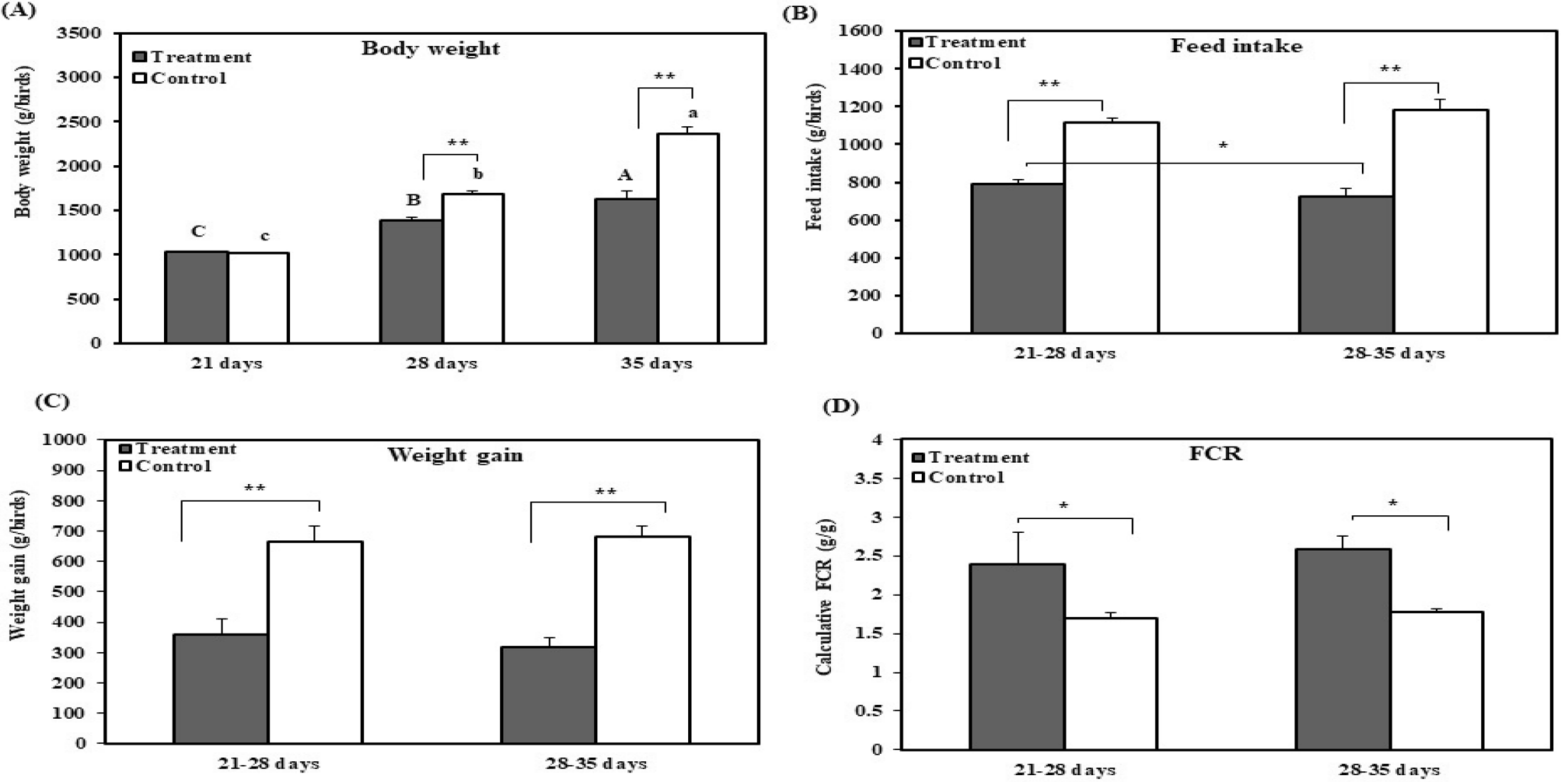
Effect of chronic heat stress on the growth performance of broilers (n = 8). (**A**) Body weight of control and treatment groups, (**B**) feed intake of control and treatment groups, (**C**) weight gain in control and treatment groups, (**D**) feed conversion ratio (FCR) in control and treatment groups. ^A–C^Different superscripts show significant differences (P < 0.05) among different time points in treatment groups. ^a–d^Different superscripts exhibit significant differences (P < 0.05) among different time points in the control group. Asterisk (*) represents statistical difference between control and treatment groups, *P < 0.05, **P < 0.01.

The prerequisite for broiler growth performance is comfortable temperature. Therefore, we analysed the effect of chronic heat stress on broiler growth performance by measuring total body weight as well as feed intake of 28 and 35 days old broiler. The effects of chronic heat stress on the growth performance of broilers are shown in Fig. 1. The total body weight of the broilers from the treatment group was significantly (*p* < 0.01) lower on days 28 and 35 compared to control (Ctrl) group. Total body weight significantly increased (*p* < 0.01) with the age of broilers in both groups until days 35. However, the feed intake significantly (*p* < 0.01) decreased in the treatment group at 21–28 and 28–35 days as compared with that in the Ctrl group. In addition, the amount of feed intake was significantly lower at 28–35 days than that at 21–28 days in treatment group. The weight gain reported for the treatment group was significantly (*p* < 0.01) lower than that observed for the Ctrl group at 21–28 and 28–35 days; however, no significant difference in weight gain was observed between the two stress time points (21–28 days and 28–35 days). Therefore, FCR was significantly (*p* < 0.05) higher for the treatment group than for the Ctrl group at 21–28 and 28–35 days. In the experimental period, a total of 5 broilers had died in the experimental group and 3 broilers died in the control group (data not shown).

### Effects of chronic heat stress on serum enzyme levels

Since, the level of blood serum SGPT and SGOT act as marker of liver damage, we assessed the effects of chronic heat stress on the levels of serum enzymes which are presented in Fig. S3. Chronic heat stress significantly increased SGPT and SGOT concentrations in LW group as compared with those in HW and Ctrl groups on day 35, as well as HW group at 28-day. However, there was no significant difference in SGPT and SGOT levels observed between HW and Ctrl groups on day 28.

### Effects of chronic heat stress on intestinal histology

As heat stress cause morphological damage and disorganization to broiler small intestinal epithelial cells, we examined H&E stained of different intestinal sections of broilers to determine the effects of chronic heat stress on intestine (Fig. S4). The histology results revealed autolysis in different sections of the small intestinal villi in response to heat stress. The autolysis was higher in all sections of the small intestine from LW group than that reported in HW and Ctrl groups.

### Effects of chronic heat stress on protein expression

**Figure 2:**
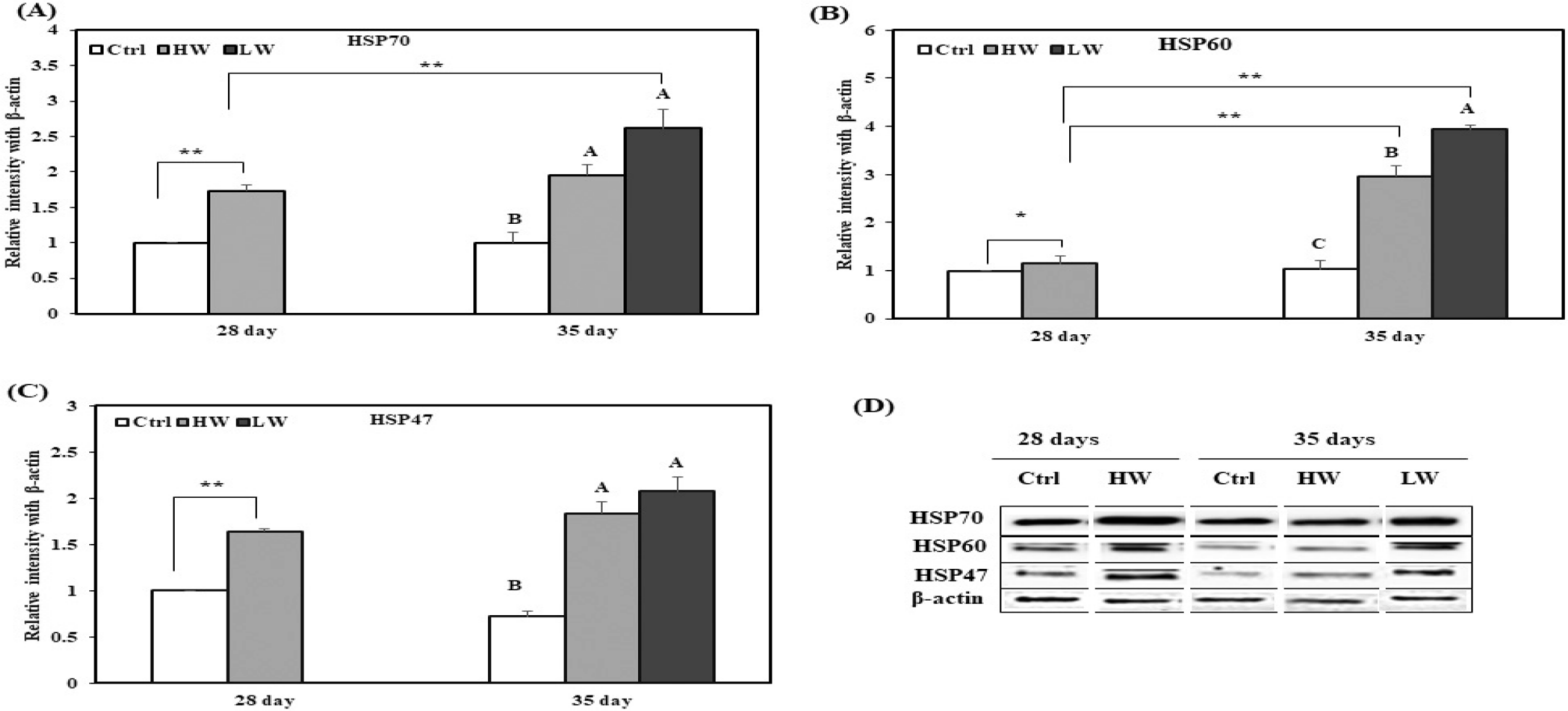
Effect of chronic heat stress on the protein expression in the duodenum of broilers (n = 8). (**A**) Mean relative expression of HSP70, (**B**) mean relative expression of HSP60, (**C**) mean relative expression of HSP47, (**D**) western blot band analysis (cropped from same gel). ^A–C^Different superscripts show significant differences (P < 0.05) among different groups on day 35. Asterisk (*) represents statistical difference between different treatment groups on day 28 and 35, *P < 0.05, **P < 0.01. We did not use the original blot in the manuscript, because it is very difficult to find out appropriate blot from the fuller length blot. The complete blots of the duodenum are presented in supplementary materials “Figure S9”.

HSPs are vitally biological molecules responsible to protect the living organism from heat stress. This ancient process is similar in all living organisms. Therefore, we evaluated the effects of two different durations of chronic heat exposure on levels of HSP70, HSP60, and HSP47 in the broiler duodenum, jejunum, and ileum by western blotting. The expression levels of HSP70, HSP60, and HSP47 in the duodenum increased on day 28 in HW group compared to the Ctrl group. On day 35, HSP70 and HSP47 levels were higher in HW and LW groups than the Ctrl group, and HSP60 expression was significantly different among HW, LW, and Ctrl groups (Fig. 2). In addition, HSP70 expression in LW group on day 35 was significantly higher than that in HW group on day 28. Moreover, HSP60 expression in HW and LW groups on day 35 was significantly higher than that reported HW group on day 28.

**Figure 3:**
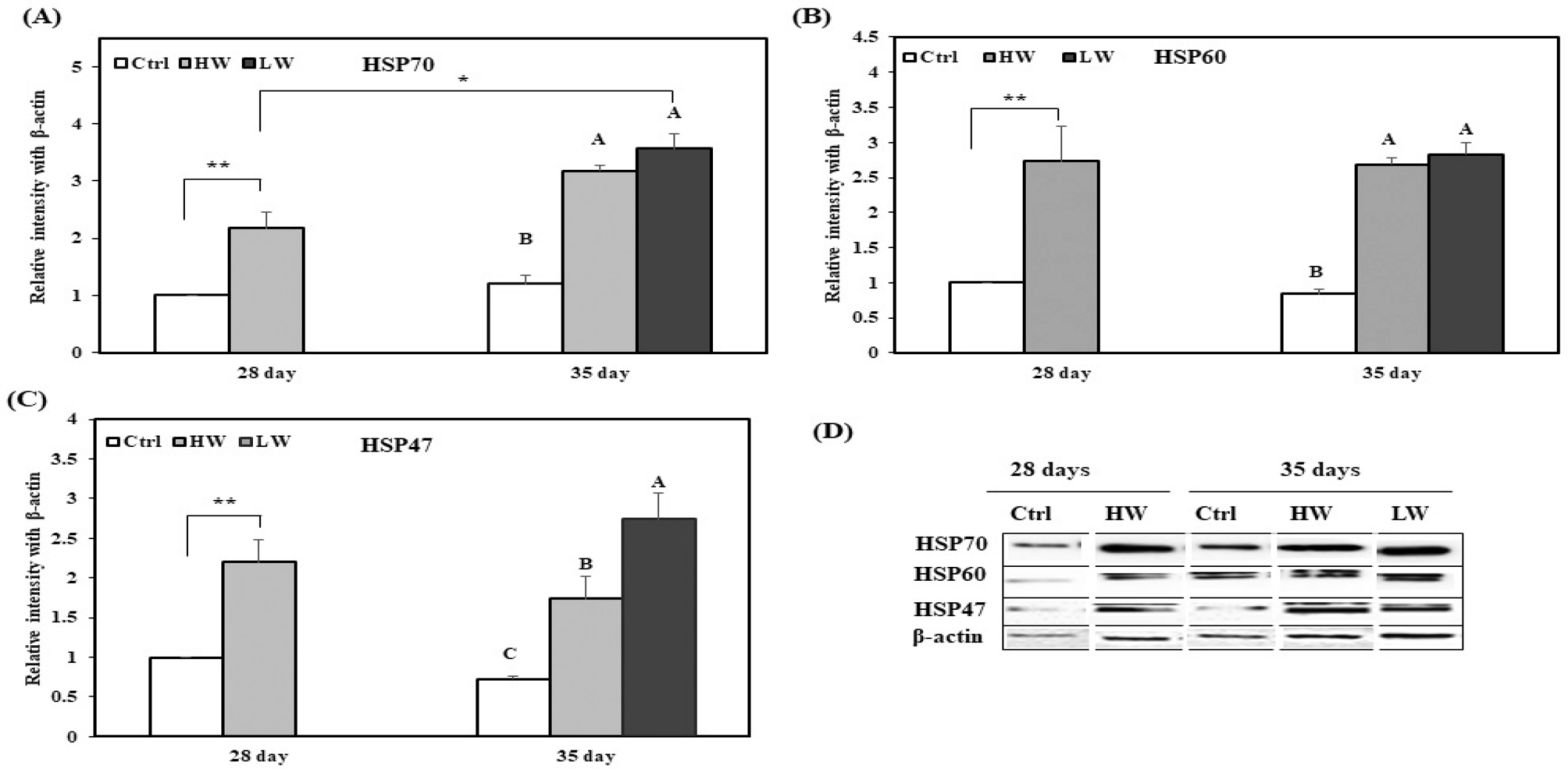
Effects of chronic heat stress on the protein expression in the jejunum of chicken (n = 8). (**A**) Mean relative expression of HSP70, (**B**) mean relative expression of HSP60, (**C**) mean relative expression of HSP47, (**D**) western blot band analysis (cropped from same gel). ^A–C^Different superscripts show significant differences (P < 0.05) among different groups on day 35. Asterisk (*) represents statistical difference between different treatment groups on day 28 and 35, *P < 0.05, **P < 0.01. We did not use the original blot in the manuscript, because it is very difficult to find out appropriate blot from the fuller length blot. The complete blots of the jejunum are presented in supplementary materials “Figure S9”.

The jejunal levels of HSP70, HSP60, and HSP47 on day 28 were higher in HW group than in the Ctrl group; on day 35, HSP70 and HSP60 expression increased in HW and LW groups as compared with that in the Ctrl group, but HSP47 expression was significantly different among the three groups (Fig. 3). Furthermore, HSP70 expression in LW group on day 35 was significantly higher than that in HW group on day 28.

**Figure 4:**
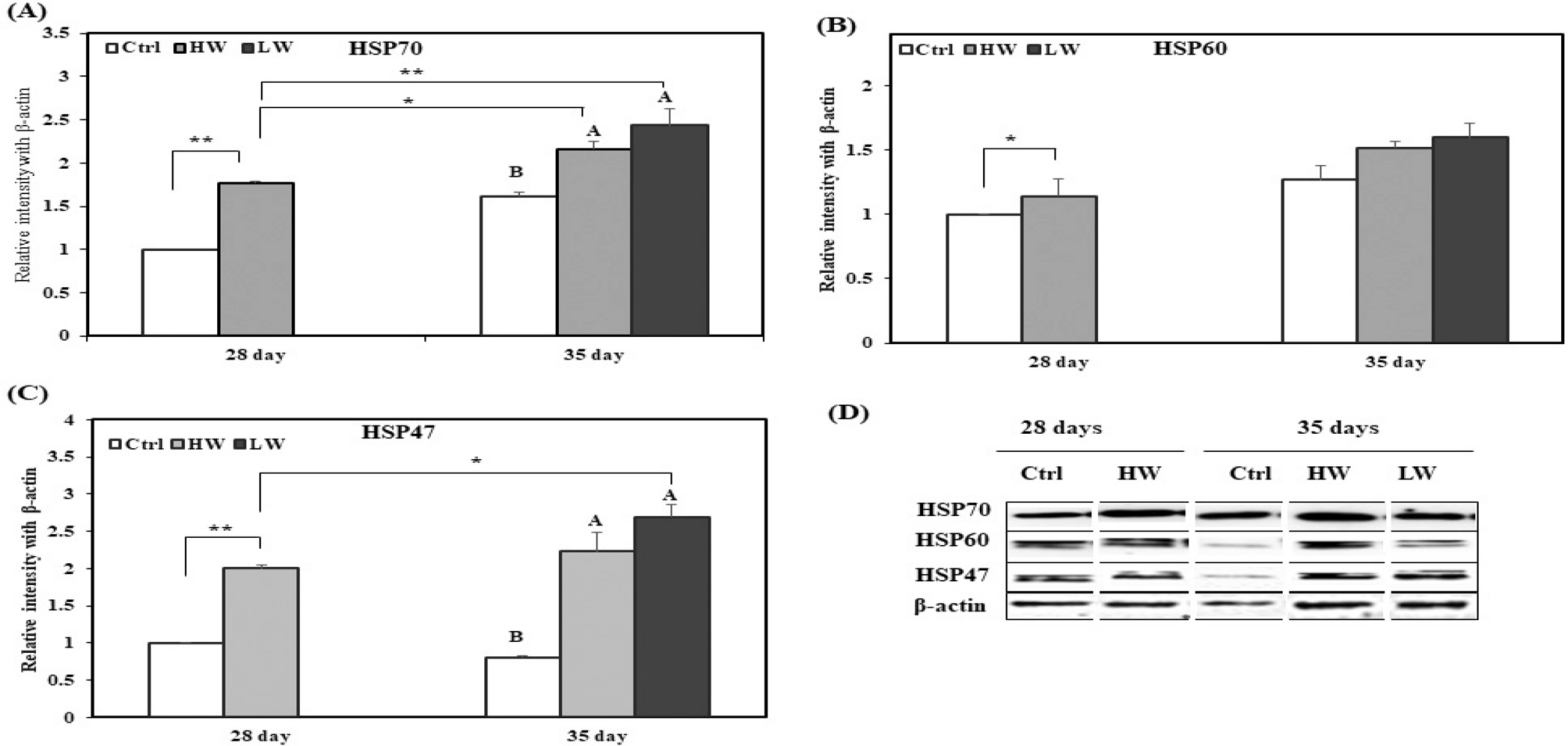
Effects of chronic heat stress on the protein expression in the ileum of chicken (n = 8). (**A**) Mean relative expression of HSP70, (**B**) mean relative expression of HSP60, (**C**) mean relative expression of HSP47, (**D**) western blot band analysis (cropped from same gel). ^A–C^Different superscripts show significant differences (P < 0.05) among different groups on day 35. Asterisk (*) represents statistical difference between different treatment groups on day 28 and 35, *P < 0.05, **P < 0.01. We did not use the original blot in the manuscript, because it is very difficult to find out appropriate blot from the fuller length blot. The complete blots of the ileum are presented in supplementary materials “Figure S9”.

The protein expression levels of HSP70, HSP60, and HSP47 in the ileum are shown in Fig. 4. The levels of HSP70, HSP60, and HSP47 on day 28 were higher in HW group than in the Ctrl group, whereas HSP70 and HSP47 levels increased in HW and LW groups as compared with those in the Ctrl group on day 35. In addition, HSP70 expression in HW and LW groups on day 35 was significantly higher than that reported on HW group at day 28. HSP47 expression in LW group on day 35 was significantly higher than that reported in HW group on day 28.

### Effect of chronic heat stress on gene expression

HSPs gene expression contribute in response by mRNA transcription during chronic heat stress. To analyse the effects of chronic heat stress on the expression levels of the genes encoding HSP70, HSP60, IL-10, IL-6, and TNF-α, RT-qPCR was conducted. The duodenal mRNA expression levels of HSP70, HSP60, and IL-10 on day 28 were significantly higher in HW group than in the Ctrl group. On day 35, the mRNA levels of HSP60 and IL-10 increased in LW group as compared to those in HW and Ctrl groups, but HSP70 mRNA level was significantly different among HW, LW, and Ctrl groups (Fig. S5). The mRNA levels of IL-6 and TNF-α were significantly lower on day 28 in HW group than in the Ctrl group, while TNF-α level decreased in LW group as compared with that in HW and Ctrl groups on day 35; IL-6 expression level was significantly different between the three groups. Further, the expression of HSP60 and IL-10 in the LW group on day 35 was significantly higher than that reported in HW group on day 28. TNF-α expression in HW group on day 35 was significantly different from that reported in HW group on day 28.

The jejunal mRNA expression levels of HSP70, IL-6, and TNF-α on day 28 were significantly higher in HW group compared to the Ctrl group. On day 35, LW group showed higher levels of HSP70, HSP60, and IL-10 mRNAs than the Ctrl group (Fig. S6). The mRNA expression levels of TNF-α on day 35 were significantly lower in LW group than in HW and Ctrl groups. In addition, the expression of IL-10 mRNA on day 35 in LW group was significantly higher than that reported in HW group on day 28. IL-10 and TNF-α mRNA expression in HW and LW groups on day 35 was significantly different from that reported in HW group on day 28.

The mRNA expression levels of HSP70, HSP60, and IL-10 in the ileum on day 28 were significantly higher in HW group than in the Ctrl group. On day 35, the expression level of HSP60 mRNA was significantly upregulated in HW and LW groups as compared with that in the Ctrl group, but HSP70 and IL-10 mRNA levels were significantly different among HW, LW, and Ctrl groups (Fig. S7). The mRNA expression of IL-6 and TNF-α was significantly downregulated on day 28 in HW group as compared with that in the Ctrl group. On day 35, IL-6 and TNF-α levels decreased in LW group as compared with that in the Ctrl group. In addition, the expression of HSP70 and IL-6 in LW group on day 35 was significantly different from that reported in HW group on day 28. Furthermore, HSP60 and IL-10 expression levels in HW and LW groups on day 35 were significantly different from those in HW group on day 28.

### Effect of chronic heat stress on the relationship between HSPs and cytokine genes

The relationship between expression of the small intestinal genes encoding HSPs (HSP70 and HSP60) and cytokines (IL-10, IL-6, and TNF-α) on day 28 and 35 between Ctrl and different heat treatment groups was investigated using the Pearson’s correlation. We performed this analysis after confirming the hypothesis of normality, linearity, and homoscedasticity. Our result revealed the complex outlines of the significant relationship between targeted genes and cytokines. For the duodenum (Table 1), the Ctrl group showed a significant association between HSP60 and IL-6 as well as TNF-α on day 28. HSP70 and IL-6 also showed a significant association in HW group on day 28. Moreover, the association between HSP70 and IL-10 was significant on day 35 in the Ctrl group. We detected a significant association between IL-6 and TNF-α on day 35 in HW group.

**Table 1:** Association among mRNA expression of heat shock proteins (HSP70 and HSP60), pro-inflammatory cytokines (IL-6 and TNF-α), and an anti-inflammatory cytokine (IL-10) in the duodenum from different groups (n = 8).

	HSP70	HSP60	IL-10	IL-6
**28-day, Ctrl**				
HSP60	0.26114			
IL-10	− 0.49691	0.39627		
IL-6	0.39440	0.97309**	0.38686	
TNF-α	0.29662	0.90929*	0.05043	0.82905
**28-day, HW**				
HSP60	0.73200			
IL-10	0.39908	0.43456		
IL-6	− 0.94621*	− 0.72740	− 0.47611	
TNF-α	0.59260	0.05022	0.35330	− 0.71369
**35-day, Ctrl**				
HSP60	− 0.33978			
IL-10	0.90015*	0.06993		
IL-6	0.75974	− 0.06088	0.83874	
TNF-α	− 0.07319	− 0.27975	− 0.11290	− 0.30323
**35-day, HW**				
HSP60	− 0.37566			
IL-10	0.35595	0.52411		
IL-6	0.75551	− 0.87531	− 0.27396	
TNF-α	0.80129	− 0.83132	− 0.22709	0.99641**
**35-day, LW**				
HSP60	− 0.05311			
IL-10	0.17627	0.52702		
IL-6	− 0.22564	− 0.70862	− 0.63935	
TNF-α	− 0.42789	0.17487	− 0.72498	0.16239

The table shows R values of parametric correlation analysis (Pearson) for HSP70, HSP60, IL-6, TNF-α, and IL-10 in different groups on day 28 and 35. Correlation is significant at **P < 0.01 and *P < 0.05.

Analysis of the jejunum (Table 2) revealed a significant association between HSP60 and IL-10 and TNF-α on day 28 in the Ctrl group. IL-10 also showed a strong association with TNF-α in the Ctrl group on day 28. The association between HSP60 and TNF-α was significant on day 35 in the Ctrl group. Furthermore, we found a significant association between HSP70 and TNF-α in HW and LW groups on day 35.

**Table 2:** Association among mRNA expression of heat shock proteins (HSP70 and HSP60), pro-inflammatory cytokines (IL-6 and TNF-α), and an anti-inflammatory cytokine (IL-10) in the jejunum of different groups (n = 8).

	HSP70	HSP60	IL-10	IL-6
**28-day, Ctrl**				
HSP60	− 0.52781			
IL-10	− 0.49962	0.93866*		
IL-6	− 0.65041	− 0.26114	− 0.31438	
TNF-α	− 0.61643	0.89439*	0.98239**	− 0.17015
**28-day, HW**				
HSP60	0.20805			
IL-10	0.33961	0.77830		
IL-6	0.68936	0.20149	0.63733	
TNF-α	− 0.01611	− 0.47933	− 0.31694	0.35097
**35-day, Ctrl**				
HSP60	0.76874			
IL-10	− 0.01780	0.48138		
IL-6	− 0.01780	0.35873	0.59413	
TNF-α	0.71944	0.90411*	0.18937	0.05358
**35-day, HW**				
HSP60	− 0.64656			
IL-10	− 0.81560	0.91586		
IL-6	0.65508	− 0.11107	− 0.50001	
TNF-α	− 0.99658**	0.60286	0.76802	− 0.63561
**35-day, LW**				
HSP60	0.04098			
IL-10	0.51301	− 0.25486		
IL-6	− 0.55964	− 0.17552	0.41625	
TNF-α	0.84820*	0.52926	0.14611	− 0.70367

The table shows R values of parametric correlation analysis (Pearson) for HSP70, HSP60, IL-6, TNF-α, and IL-10 in different groups on day 28 and 35. Correlation is significant at **P < 0.01 and *P < 0.05.

In the ileum of the Ctrl group (Table 3), a significant association of HSP70 and HSP60 with IL-10 was observed on day 28. HSP60 was also strongly associated with IL-10 in the Ctrl group on day 28. In the HW group, HSP70 had a significant association with IL-10 and TNF-α on day 28 and HSP70 and HSP60 showed a strong association on day 35.

**Table 3:** Association among mRNA expression of heat shock proteins (HSP70 and HSP60), pro-inflammatory cytokines (IL-6 and TNF-α), and an anti-inflammatory cytokine (IL-10) in the ileum of different groups (n = 8).

	HSP70	HSP60	IL-10	IL-6
**28-day, Ctrl**				
HSP60	0.94159*			
IL-10	0.91454*	0.91775*		
IL-6	0.26352	− 0.05336	− 0.01333	
TNF-α	0.68951	0.44752	0.38007	0.83374
**28-day, HW**				
HSP60	− 0.53355			
IL-10	0.97259**	− 0.62019		
IL-6	0.39067	− 0.04978	0.18685	
TNF-α	0.93252*	− 0.56846	0.85117	0.66499
**35-day, Ctrl**				
HSP60	0.65570			
IL-10	0.19980	− 0.16643		
IL-6	− 0.48212	− 0.32993	0.01970	
TNF-α	0.49315	0.21292	− 0.43809	0.15706
**35-day, HW**				
HSP60	0.90149*			
IL-10	0.85584	0.77983		
IL-6	0.64139	0.47868	0.68157	
TNF-α	0.62986	0.83238	0.31448	0.04425
**35-day, LW**				
HSP60	− 0.40745			
IL-10	0.21202	0.54849		
IL-6	− 0.63428	0.70764	0.39745	
TNF-α	− 0.38362	0.09417	0.22674	0.75557

The table shows R values of parametric correlation analysis (Pearson) for HSP70, HSP60, IL-6, TNF-α, and IL-10 in different groups on day 28 and 35. Correlation is significant at **P < 0.01 and *P < 0.05.

## Discussion

As expected, we observed progressive alterations in the morphology of the small intestine of broiler, consistent with an increase in the levels of SGPT and SGOT during chronic heat stress. As a consequence, it’s growth performance drastically declined^5^. In addition, Chronic heat stress impairs the growth performance, intestinal structure. Previous study reported that heat stress leading to significantly decreased feed intake and growth performance as well as increased FCR of broiler^37^, inducing intestinal injury, dysfunction of nutrient metabolism^38,39^. This alteration in the intestinal epithelium was associated with the decline in growth performance, changes in the expression of HSPs, and inflammation. Before reported the chronic heat stress-induced attenuation of the innate immune system^24^. In the present study, it had been observed changes in the biochemical parameters, HSPs, and pro- and anti-inflammatory cytokines in response to chronic heat stress and their effects on the immune system^40^. Heat stress affected the liver function and reduced bile acid content^41^. In the absence of bile acid content, the intestinal epithelial cells lose their integrity with respect to the bacterial load^42^. Heat stress changes the intestinal morphology by inducing autolysis and sloughing the intestinal villi^43,44^. It has been reported that chronic heat stress damaged the 35-day aged broiler intestinal morphology^5^. In addition, heat stress leading to intestinal ischemia and impairments of intestinal morphology in broiler^45^. Intestinal integrity regulates the intestinal tight junction, as well as influences the immune system^46^. It has been reported that the status of the immune system is dictated by cytokines expression, which impairs the growth performance^47^. The results of the growth performance of broilers in our study are in line with the previous results, as we observed that decreases broilers body weight, along with increased cytokines expression in heat stress group than the control group.

Exposure to chronic heat stress for different time points results in the overexpression of HSP70, HSP60, and HSP47 in different sections of broiler small intestine. HSP70 is known to induce protein folding and prevent protein aggregation^48^. A previous study found that HSP70 prevents cell death through its effects on mitochondrial permeability^49,50^. Our study result is in line with that reported in a previous study, wherein piglets from lower body weight group showed higher expression of HSP70 in different sections of their intestine and HSP70 exerted protective effects against stress^51^. HSP60 has a crucial role in the protection of different visceral organs of broilers and rats^52–54^. However, there is no study that has evaluated the protective function of HSPs in different sections of the small intestine of broilers. According to our results, the expression of HSP60 has significantly increased in the heat stressed broilers compared to the control broiler. This result suggests that HSP60 may play a protective role in the intestine against chronic heat stress. Previous study reported that HSP60 is a mitochondrial stress protein, which showed the response of stress and inflammation, as well as diminution the inflammation^55^ and prevent the protein denaturation^56^. The result of the present study demonstrates the increase in the expression of HSP47 after heat stress exposure. However, previous study reported that there is no relation between HSP47 and pro- and anti-inflammatory cytokine gene expression^23^. Although, the expression of HSP47 has a relation to the level of collagen damage in response to heat stress^57,58^. In this study, the expression of HSP47 during chronic heat stress is consistent with the previous study. Therefore, we recommended that chronic heat stress regulates HSP expression levels and the growth performance of broiler.

In the present study, the analysis of relationship between HSPs (HSP70 and HSP60) and pro- and anti-inflammatory cytokines (IL-6, TNF-α, and IL-10) revealed the role of specific HSPs and cytokines during inflammation and immune response in the different sections of the small intestine. However, chronic heat stress suppresses the expression of pro-inflammatory cytokines and promotes the expression of anti-inflammatory cytokines, resulting in the activation of the adaptive immunity^59,60^. One hypothesis of our study is that HSP70 and HSP60 interact with pro- and anti-inflammatory cytokines (IL-6, TNF-α, and IL-10) to reduce inflammation by strengthening the immunity of the small intestine. It has been demonstrated that HSP70 impedes the expression of pro-inflammatory cytokines^61,62^ and accelerates the production of anti-inflammatory cytokines^63^. These reports are in line with our observation that HSP70 shows a relationship with pro-inflammatory cytokines (IL-6 and TNF-α) and anti-inflammatory cytokines (IL-10) in the small intestine of broiler in response to chronic heat stress. HSP60 responses to pro- and anti-inflammatory signals depending on its concentration and specific epitope^64^. Nonetheless, anti-inflammatory cytokines suppress the expression of pro-inflammatory cytokines^65^. For instance, IL-10 inhibits the expression of IL-6 and TNF-α in stimulated macrophages^66^.

In conclusion, we analyzed the protein expression of HSPs, as well as the relation between HSPs and Pro- and anti-inflammatory cytokines of the different sections of broiler small intestine, those broilers had been treated with chronic heat stress. Our results revealed that chronic heat stress may severely affect the intestinal immunity of broilers, thereby disturbing their growth performance and intestinal morphology, as well as increasing HSP expression. Moreover, the broilers activate adaptive immunity in the intestine in response to chronic heat stress (Fig. S8), consequently, their heat adaptive capacity has increased.

## Materials and methods

### Ethics statement

In this study, all experiments were performed in accordance with relevant guidelines and regulations of Jeonbuk National University. All animal care and experiments were approved by the Animal Experiment Administration Committee of Jeonbuk National University (approval number: CBNU2018-097). All work was accomplished to reduce the distress of broilers throughout the experiments and carried out according to that protocol.

### Broiler management and experimental design

The broilers (n = 8 replicates) used in this study were raised in cages (dimension of each cage: length × width × height, 190 × 120 × 50 cm) in an environmentally control house with ad libitum commercial feed (Table S1) and fresh water until experiment completion. A total of 240 1-day-old broiler chicks (Ross) were obtained from a commercial hatchery in Iksan, Republic of Korea. These chicks were raised for up to 20 days in 16 cages (15 chicks/cage) in the aforementioned farmhouse. The brooding temperature was 33 °C for first 2 days, and then gradually decreased to 26 °C by 21 days (2 °C/week). In the control (Ctrl) group, the temperature was gradually decreased to 22 °C by 35 days. Previous study reported that broiler gain significantly higher body weight at 21 to 35 day of age^67^. Consequently, we selected 21 to 35 days aged broiler for our study. Therefore, the incubation temperature for the heat stress group was 34 °C from day 21 to 35. The relative humidity was controlled at around 50% in both control and treatment groups. After 20 days of acclimation, the broilers were divided into two temperature groups (Ctrl and heat stress) with eight replications per group (Fig. S1). Each replication included 10 birds of approximately same body weight. Heat stress reduces body weight and immunity^68,69^. Consideration this fact, in the treatment group, all broiler was subdivided in the lower and higher body weight group based on their body weight during sampling^70^. On day 28, the body weight of broiler from the Ctrl group was > 1500 g. The treatment group was subdivided into heavy body weight (HW) group (1000–1500 g) and light body weight (LW) group (< 1000 g) (Fig. S2A,B). On day 35, the body weight of each broiler of Ctrl group was > 2000 g. In the treatment group, the body weight in the HW group was 1500–2000 g/broiler, and < 1500 g/broiler was in the LW group. In the 28 days, we skipped the LW group, because only 2% of broilers were in this group. In contrast, in 35 days, we collected the sample from both HW (67.56%) and LW (32.43%) (Fig. S2C) group for the higher number of broiler.

### Sample collection

At 28 and 35 days of age, we measured the body weight and cumulative feed intake of broilers from each replication, thereafter we calculate the weekly weight gain and feed conversion ratio (FCR). A total of eight broilers were randomly selected from each group and blood was collected from the wing veins. After that, immediately sacrificed by cervical dislocation and instantly had been collected the duodenum, jejunum, and ileum. The intestinal content was washed with phosphate-buffered saline (PBS) without causing damage to the tissues. All the dissected intestinal parts were kept in liquid nitrogen for a moment and then stored at − 80 °C until western blot and reverse-transcription quantitative polymerase chain reaction (RT-qPCR) analyses. The serum was collected by centrifugation of blood samples at 3000×*g* for 15 min. The serum samples were stored at − 80 °C until analysis.

### Growth parameters of broilers

The body weight gain and FCR were calculated from total body weight and feed intake on day 28 and 35 of treatment as follows:

$$\text{Body weight gain} = \text{Final body weight } - \text{Initial body weight}$$

$${\text{FCR = }}\frac{{{\text{Total}}\;{\text{feed}}\;{\text{intake}}}}{{{\text{Total}}\;{\text{body}}\;{\text{weight}}}}$$

### Serological analysis

Serum glutamic pyruvic transaminase (SGPT) and serum glutamic-oxaloacetic transaminase (SGOT) levels of both groups (n = 8) were measured using commercial kits (Asan Pharmaceutical, CO. Ltd., Seoul, Republic of Korea), as per the manufacturer’s instructions.

### Histopathological analysis

After collection, all intestinal samples were immediately fixed with 4% (v/v) paraformaldehyde and embedded in paraffin using an autoprocessor (Thermo Scientific, Waltham, USA). Each paraffin embedded sample was sectioned (5 μm thickness) and stained with haematoxylin and eosin (H&E)^71^. The histopathological variations in the intestine were observed using a Leica DM2500 microscope (Leica Microsystems, Wetzlar, Germany) at 100× magnification.

### Total protein extraction and western blotting

The intestinal samples were ground in liquid nitrogen and the obtained powder was mixed (100 mg sample) with 1 mL of radioimmunoprecipitation assay (RIPA) buffer (150 mM sodium chloride, 1% Triton X-100, 0.1% sodium dodecyl sulphate [SDS], 1% sodium deoxycholate, 50 mM tris–HCl pH 7.5, 2 mM ethylenediaminetetraacetic acid [EDTA]), and protease inhibitor (Invitrogen, NY, USA) for lysis and extraction of proteins. The concentration of the extracted protein was measured with the DC protein assay kit (Bio-Rad, California, USA). The samples were mixed with a sample buffer (Cat #EBA-1052, Daejeon, Republic of Korea) and heated at 95 °C for 10 min and then cooled to 4 °C. These protein samples were separated on suitable acrylamide gels, and the separated bands were electrophoretically transferred onto polyvinylidene fluoride (PVDF) membranes (Cat #162-0177, Bio-Rad, USA). These membranes were blocked with 5% skim milk in TBST (0.05% Tween 20, 100 mM Tris–HCl, and 150 mM sodium chloride [NaCl], pH 7.5) at room temperature (25 °C) for 1 h and then incubated with specific primary antibodies at 4 °C for overnight. After washing these membranes with TBST for 10 min (three washes), they were probed with an horseradish peroxidase (HRP)-conjugated secondary antibody for 1 h at room temperature. A chemiluminescent substrate (Invitrogen NY, USA) and iBright CL1000 (Thermo Fisher Scientific, Waltham, USA) were used for protein band visualisation and quantification. The primary antibodies used were mouse monoclonal HSP70, HSP60, and HSP47 (Enzo Life Science, NY, USA) (1:2500). The expression of protein was normalised using a primary antibody to β-actin (Santa Cruz Biotechnology, Texas, USA) (1:2500). The secondary antibody used was goat anti-mouse IgG (Enzo Life Science, USA) (1:5000).

### Total RNA extraction and RT-qPCR analysis

Total RNA was extracted from different parts of broiler small intestine with the Trizol reagent (Invitrogen, USA) according to the manufacturer’s instructions. The concentration and purity of RNA was determined using a Nanodrop spectrometer (Invitrogen, NY, USA) at 230 nm and 260/280 nm, respectively. One microgram of RNA was used for cDNA synthesis by reverse-transcription using iScript cDNA synthesis kit (BIO-RAD, USA) according to the manufacturer’s instructions. cDNA was amplified using SYBR green Supermix (BIO-RAD, USA) on a CFX96 Real-Time PCR Detection System (Bio-Rad, USA). Specific primers were used for the inspected genes (Table S2) to study the relationship between HSP70/HSP60 and pro- and anti-inflammatory cytokines. Amplification was started at 95 °C for 30 s, followed by 40 cycles of 95 °C for 5 s and 58 °C for 5 s. The melting temperature was 65 °C to 95 °C for 5 s. Relative mRNA fold change was calculated using the ΔΔCt method, and glyceraldehyde 3-phosphate dehydrogenase (GAPDH) was used as the reference gene. RT-qPCR results were calculated using the ΔCt value (Ct target gene − Ct GAPDH). Relative gene expression was obtained using the ΔΔCt method. The Ctrl group was used for comparing gene expression. Fold induction was calculated as 2^−ΔΔCt^, wherein 2^−ΔΔCt^ is the relative gene expression^72^.

### Statistical analysis

All data analyses were performed using SAS version 9.4 (SAS Institute Inc., Cary, NC, USA). Data are presented as mean ± standard error (SE). The t-test was used to analyze the effect of chronic heat stress on growth performance parameters, as well as gene and protein expression of different groups in different periods of time. The protein and gene expression of 35-days different groups, as well as body weight in different periods of time were analyzed by one-way analysis of variance (ANOVA) followed by Duncan’s multiple range test. Correlations among different gene expression were evaluated using Pearson’s correlation coefficients. We considered p < 0.05 and p < 0.01 to represent statistical significance.

## Supplementary information

Supplementary Information.
