# Supplementary material for: Chronic heat stress regulates the relation between heat shock protein and immunity in broiler small intestine
Source: Sci Rep. 2020 Nov 2;10:18872. doi: 10.1038/s41598-020-75885-x (PMC7608671; doi:10.1038/s41598-020-75885-x)
Supplement: Supplementary file 1 — Supplementary Information. [file 41598_2020_75885_MOESM1_ESM.pdf]

Supplementary Information

Chronic heat stress regulates the relation between heat shock protein and immunity in broiler small intestine

Sharif Hasan Siddiqui[1], Darae Kang[1], Jinryong Park[1], Mousumee Khan[2], and Kwanseob Shim[1,*]

[1]Department of Animal Biotechnology, College of Agriculture and Life Sciences, Jeonbuk National University, Jeonju 54896, Republic of Korea

[2]Department of Biomedical Sciences and Institute for Medical Science, Jeonbuk National University Medical School, Jeonju, 54907, Republic of Korea

[*]Corresponding. ksshim@jbnu.ac.kr (K. S. S)

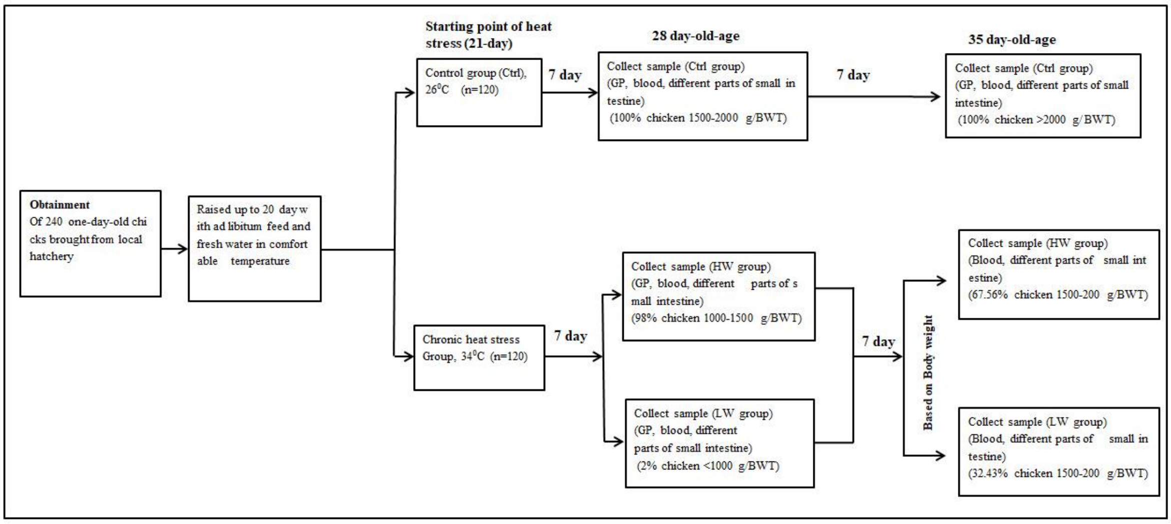

Figure S1. Graphical experimental design of the work carried out in this study.

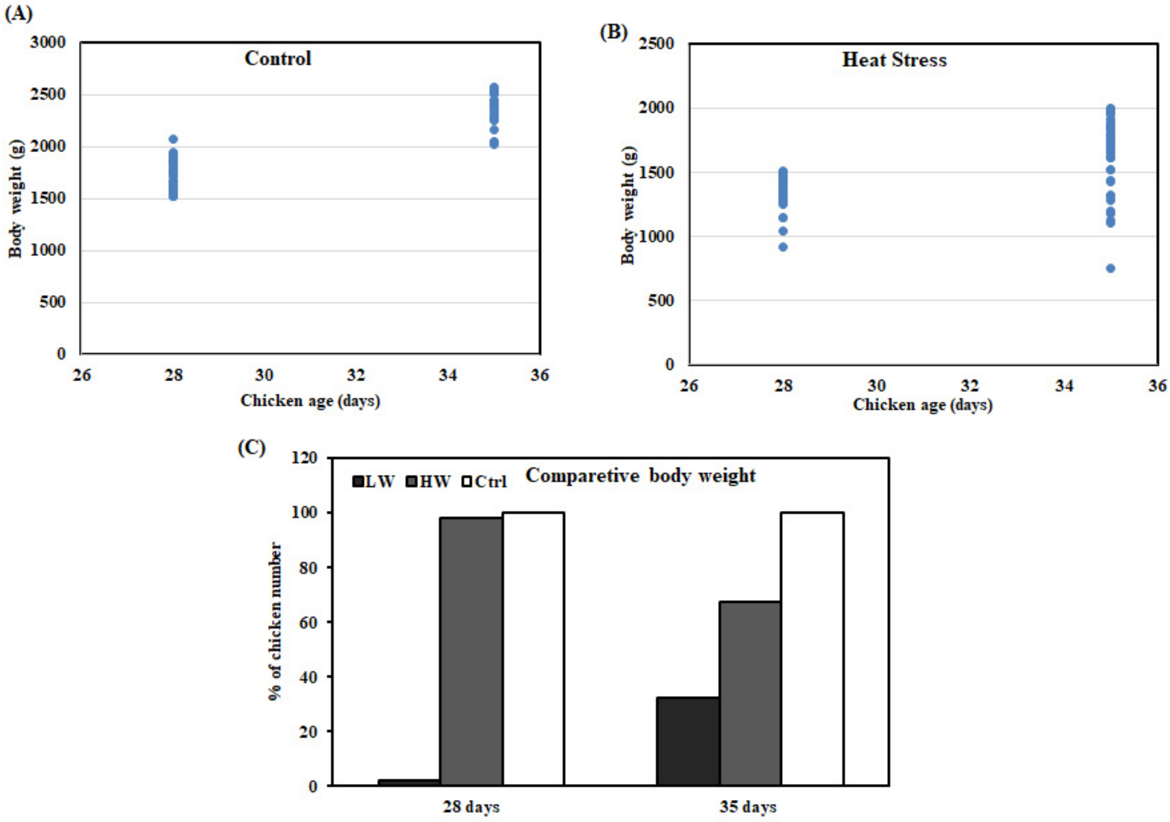

Figure S2. Effect of chronic heat stress on total body weight of broiler. (A, B) Range of body weight in the control and treatment groups on day 28 and 35, (C) chicken number (%) in each group at day 28 and 35.

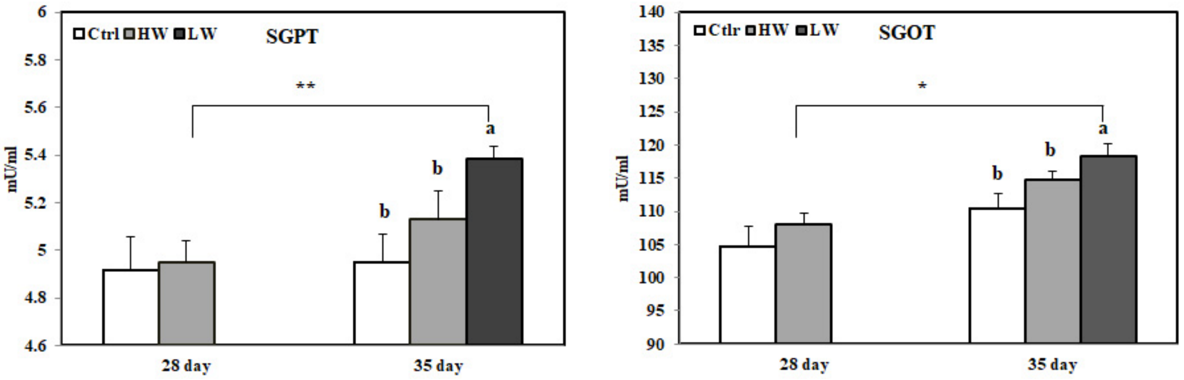

Figure S3. Levels of serum glutamic pyruvic transaminase (SGPT) and serum glutamic oxaloacetic transaminase (SGOT) upon exposure to chronic heat stress (A) and (B). [a-b]SGPT and SGOT in columns with different superscripts significantly differ ($P < 0.05$) among different groups at day 35. Asterisk (*) represents statistical difference between different treatment groups on day 28 and 35, *$P < 0.05$ and **$P < 0.01$.

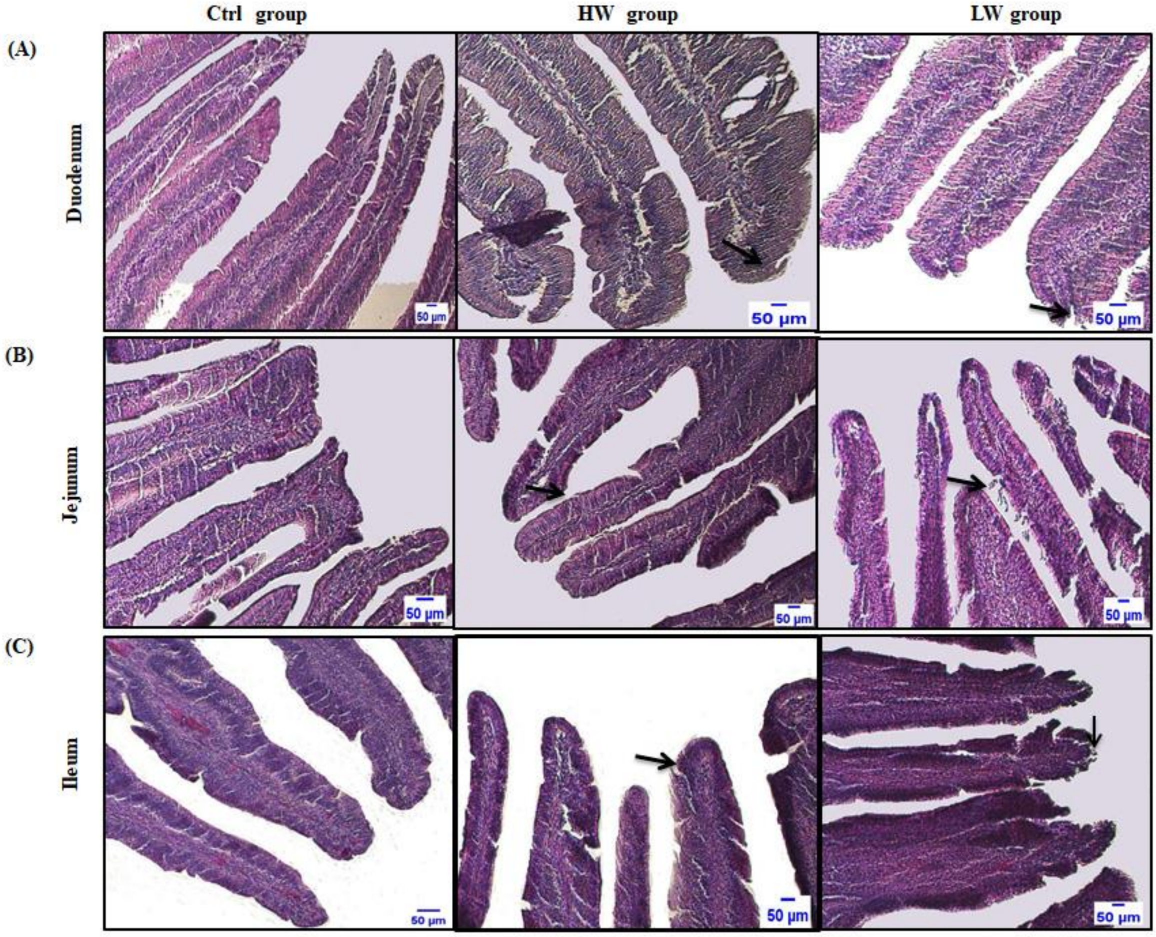

Figure S4. Effect of chronic heat stress on the morphology of the small intestinal villi of broilers from each group on day 35. (A) Morphology of duodenum villi, (B) morphology of jejunum villi, (C) morphology of ileum villi.

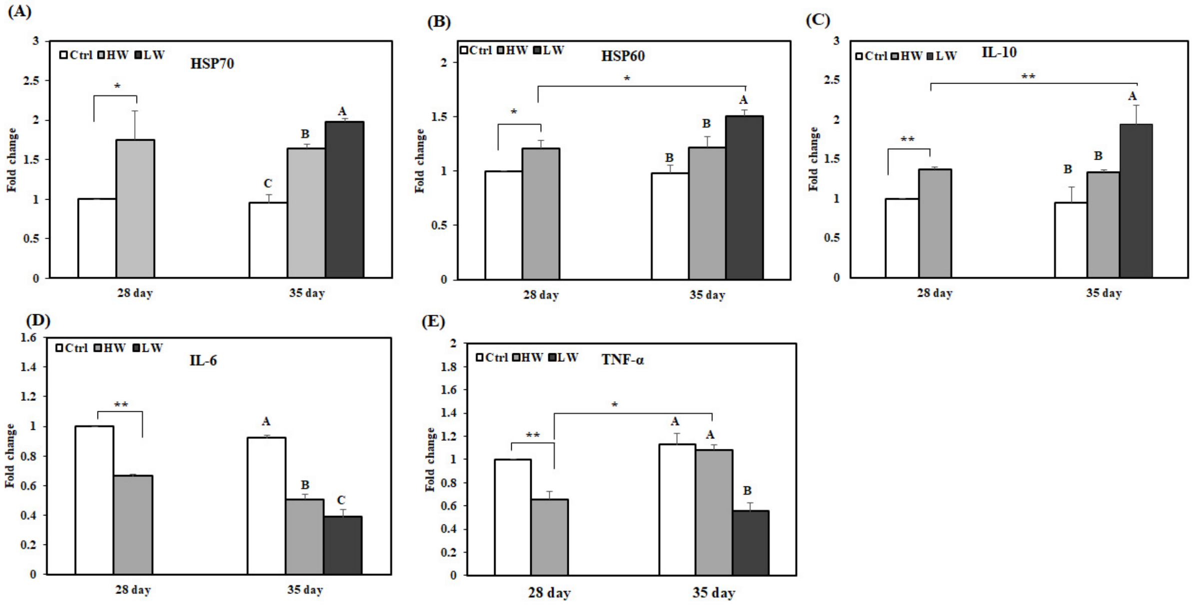

Figure S5. Effect of chronic heat stress on the mRNA expression in the duodenum of chicken. (A) Mean relative expression of HSP70, (B) mean relative expression of HSP60, (C) mean relative expression of IL-10, (D) mean relative expression of IL-6, and (E) mean relative expression of TNF-α. $^{A\text{-}C}$Different superscripts show significant differences ($P < 0.05$) among different groups at day 35. Asterisk (*) represents statistical difference between different treatment groups at day 28 and 35, *$P < 0.05$ and **$P < 0.01$.

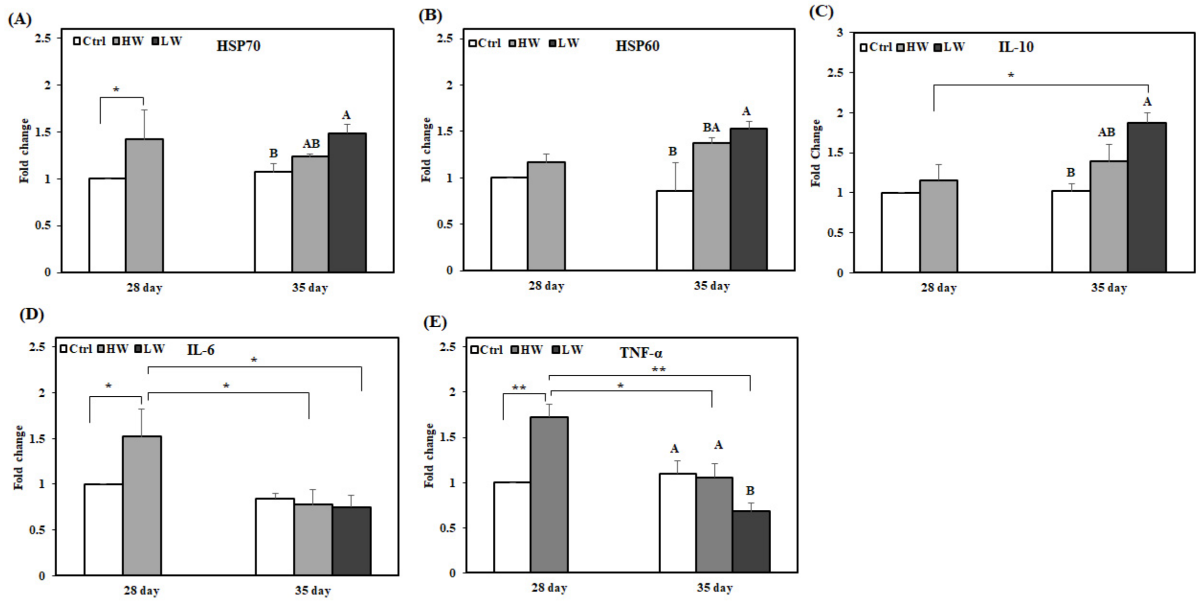

Figure S6. Effect of chronic heat stress on the mRNA expression in the jejunum of chicken. (A) Mean relative expression of HSP70, (B) mean relative expression of HSP60, (C) mean relative expression of IL-10, (D) mean relative expression of IL-6, and (E) mean relative expression of TNF-α. $^{A\text{-}B}$Different superscripts show significant differences ($P < 0.05$) among different groups at day 35. Asterisk (*) represents statistical difference between different treatment groups on day 28 and 35, *$P < 0.05$ and **$P < 0.01$.

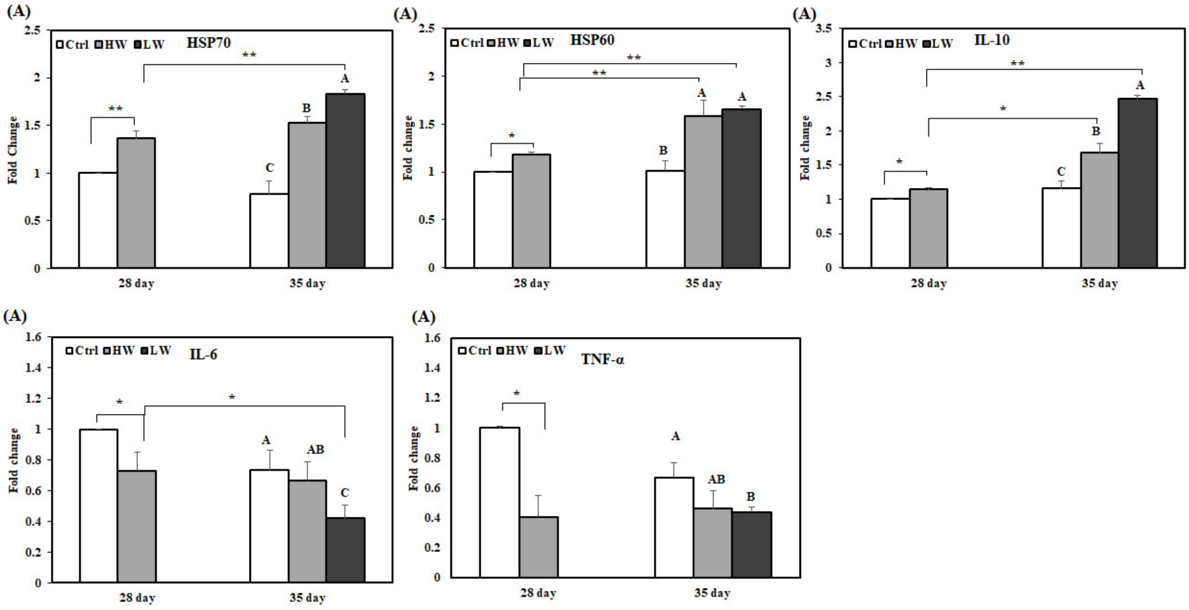

Figure S7. Effects of chronic heat stress on the mRNA expression in the ileum of chicken. (A) Mean relative expression of HSP70, (B) mean relative expression of HSP60, (C) mean relative expression of IL-10, (D) mean relative expression of IL-6, and (E) mean relative expression of TNF-α. $^{A\text{-}C}$Different superscripts show significant differences ($P < 0.05$) among different groups at day 35. Asterisk (*) represents statistical difference between different treatment groups on day 28 and 35, *$P < 0.05$ and **$P < 0.01$.

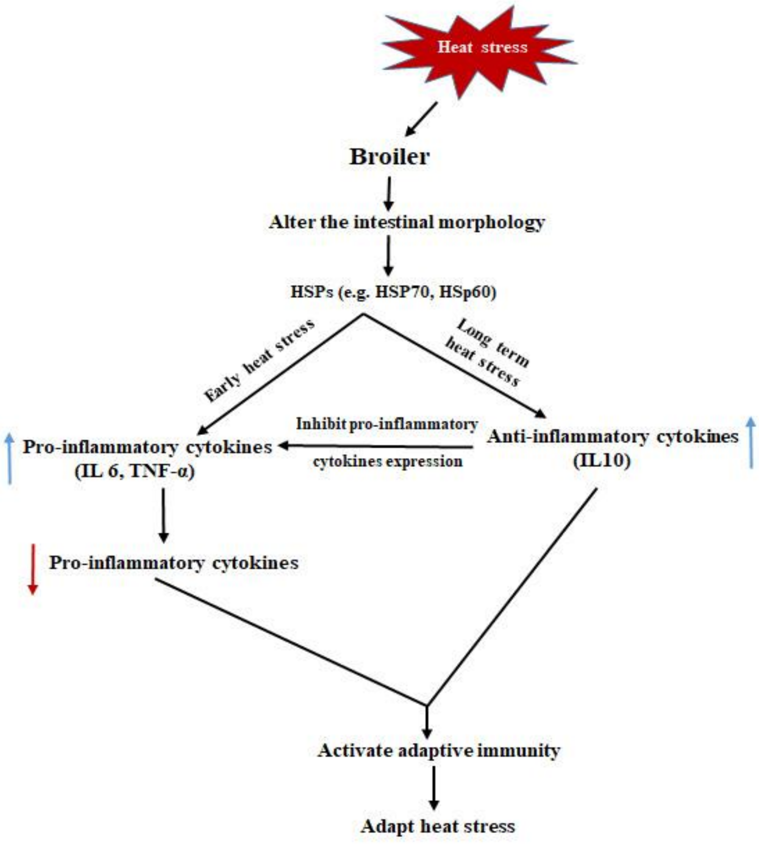

Figure S8. Schematic diagram showing the molecular mechanism underlying the activation of the adaptive immunity by HSP70 and HSP60.

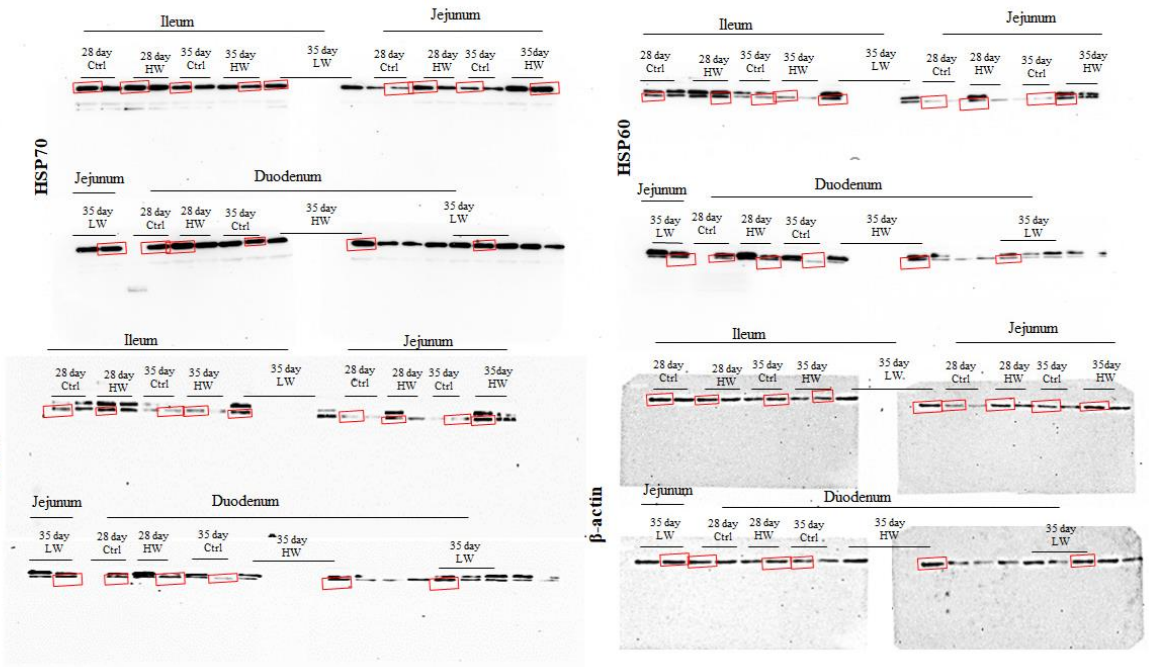

Figure S9. The western blotting band of HSP70, HSP60, HSP47, and β-actin in different sections of the broiler's small intestine.

Table S1. Chemical composition of broiler feed.

Feed's chemical Composition	Starter*	Finisher**
Crude protein	20.0%	19.0%
Crude fat	4.0%	4.0%
Calcium	0.75%	0.75%
Phosphate	0.70%	0.70%
Crude fiber	6.0%	5.5%
Crude ash	8.0%	8.0%
Met + Cys + MHA$^{\#}$	0.75%	0.65%
ME$^{\#\#}$	3.00 Mcal/kg	3.05 Mcal/kg

*Starter, 0–20 day; ** Finisher, 21–35 day; $^{\#}$Met + Cys + MHA, DL-Methionine + Cysteine + DL-Methionine hydroxyl analogue; $^{\#\#}$ ME, metabolizable energy.

Table S2. The primers for qPCR.

Primer name	Primer sequence (5′→ 3′)	Annealing temp. (°C)
HSP70	F– GGTAAGCACAAGCGTGACAATGCT<br>R–TCAATCTCAATGCTGGCTTGCGTG	55
HSP60	F– AGAAGAAGGACAGAGTTACC<br>R– GCGTCTAATGCTGGAATG	55
IL-10	F – CTGAAGGCGACGATGC<br>R – TTCCTCCTCCTCATCAGC	55
IL-6	F – CTCCTCGCCAATCTGAAGTC<br>R – CCCTCACGGTCTTCTCCATA	61
TNF-α	F–AGGCCAGATGGGAAGGGAATGAA<br>R – GAAGAGGCCACCACACGACAG	62
GAPDH	F–AGAACATCATCCCAGCGTCC<br>R–CGGCAGGTCAGGTCAACAAC	60

HSP; Heat shock protein, IL; Interleukin, GAPDH; Glyceraldehyde 3-phosphate dehydrogenase.
